# A Weighted Multidomain Score for Prioritizing Nutritional Review in Institutionalized Older Adults: Development and Internal Assessment

**DOI:** 10.3390/nu18152533

**Published:** 2026-08-04

**Authors:** Elena Moreno-Guillamont, Carmen I. Sáez-Lleó, Maria Auxiliadora Dea-Ayuela, Jose M. Soriano

**Affiliations:** 1 Sociosanitary Pharmacy Services, Universal and Public Health Department, Valencian Government, 46015 Valencia, Spain; moreno_elegui@gva.es; 2 Food & Health Lab, Institute of Materials Science, University of Valencia, 46980 Paterna, Spain; saez_carlle@gva.es; 3 Departament of Pharmacy, Faculty of Health Sciences, CEU Cardenal Herrera University, 46115 Alfara del Patriarca, Spain; mdea17@gmail.com; 4 Joint Research Unit on Endocrinology, Nutrition and Clinical Dietetics, Health Research Institute La Fe-University of Valencia, 46026 Valencia, Spain

**Keywords:** VIVA-NIPI, weighted multidomain score, nutritional review priority, institutionalized older adults, long-term care, construct concordance, sensitivity analysis

## Abstract

**Background/Objectives:** Institutionalized older adults often present overlapping functional, cognitive, nutritional, intake-related, anthropometric, and biochemical abnormalities. We developed a prespecified weighted multidomain nutritional priority score (VIVA-NIPI) to organize the urgency of comprehensive nutritional review and assessed its internal construct concordance and ranking stability. **Methods:** Routine-care data from 124 residents in 10 long-term care facilities in Valencia, Spain, collected from June 2022 to April 2024, were combined across functional–cognitive, nutritional-intake/anthropometric, and selected biochemical-indicator domains. Weights were defined by the research team from clinical and methodological judgement rather than derived through a formal expert pairwise-comparison exercise; algebraically generated consistency ratios were not interpreted as validation evidence. The reference constructs overlapped with score components and therefore assessed internal concordance rather than independent clinical validity. **Results:** The continuous score averaged 24.4 ± 12.2. The partly overlapping k-means phenotype yielded an AUC of 0.918 (95% bootstrap CI 0.870–0.958); AUCs were 0.769 for MNA-SF ≤ 11, 0.602 for intake < 75%, and 0.782 for albumin < 3.5 g/dL. These values do not establish diagnostic or prognostic performance. Sensitivity analyses showed Spearman correlations of 0.991 after removing age, 0.960 after removing MNA-SF, 0.957 for an unweighted score, 0.983 when retaining only albumin and total proteins in the biochemical domain, and 0.901 after removing that do-main. **Conclusions:** VIVA-NIPI is a preliminary weighted multidomain research score, not a clinically validated triage tool. Independent clinical adjudication or prospective outcomes, external centre-aware validation, formal stakeholder weight elicitation, missing-data modelling, and calibration of actionable thresholds are required before implementation.

## 1. Introduction

Institutionalized older adults are clinically heterogeneous and often combine functional dependency, cognitive impairment, mobility limitation, nutritional risk, biochemical alterations and reduced ability to respond to standard menus. In this context, the clinical question is not only whether a resident is vulnerable, but which resident requires nutritional intervention first and what dimension should drive that decision.

Comprehensive geriatric assessment recognizes that adverse outcomes in older people arise from interacting functional, cognitive, nutritional and social factors rather than from isolated diagnoses alone [1,2]. Nutritional screening instruments such as the Mini Nutritional Assessment and its short form are widely used in older adults [3,4], and previous work in Archives of Gerontology and Geriatrics has supported the validity of both the long and short forms in older populations [5]. However, long-term care decisions are rarely based on nutritional screening alone. Evidence from nursing-home and residential long-term care settings shows that malnutrition coexists with frailty and predicts adverse outcomes [6], is associated with falls and impaired activity [7], and remains a persistent care issue over time [8]. Functional dependency, fall risk, cognition, body composition, observed intake, biochemical reserve and feasibility of menu adaptation therefore often determine whether, when and how interventions can be implemented.

Several approaches have attempted to operationalize frailty or vulnerability through cumulative or phenotypic indices [9,10]. These approaches are useful for risk stratification, but they do not automatically translate into nutritional prioritization. A resident may have high functional dependency but preserved intake, whereas another may have moderate dependency but low intake, low albumin and low MNA-SF. These situations require a transparent decision framework that can integrate heterogeneous information while preserving clinical interpretability.

MNA-SF is a screening instrument, GLIM supplies diagnostic criteria, and frailty indices summarize vulnerability or deficit accumulation. None of these tools were designed to rank residents specifically by urgency of nutritional review while simultaneously incorporating observed intake, functional capacity, cognition, anthropometry, and biochemical reserve. The proposed framework therefore addresses prioritization rather than screening, diagnosis, or prognosis. Its primary intended output is the sequence in which residents should undergo nutritional review; possible menu adaptation, eating assistance, supplementation, and allocation of dietetic or nursing resources are downstream actions that require clinical assessment.

Multicriteria decision frameworks can make domains, subcriteria, and weighting assumptions explicit [11,12]. In this study, however, weights were selected by the research team from clinical and methodological judgement and were not derived through a formal independent expert pairwise-comparison process. The model is therefore described as a prespecified weighted multidomain score. Reciprocal matrices derived from the selected weights document the original structure only; their expected consistency is not evidence of methodological validity. The application of explicit multicriteria weighting to dietary-pattern assessment provides a relevant nutrition-related precedent [13].

Transparent multidomain scoring may be useful when long-term care teams must combine heterogeneous information, but it also introduces subjectivity, possible double-counting, missing-data dependence, and equity concerns. The present study therefore focuses on explicit score construction, internal construct concordance, and sensitivity analysis rather than claiming formal AHP development or clinical validation.

The objective is to develop and internally assess a weighted multidomain nutritional priority score for institutionalized older adults. The intended user is a multidisciplinary long-term care team, the time horizon is near-term review, and the intended action is comprehensive nutritional reassessment rather than automatic supplementation, menu prescription, or resource allocation. The score summarizes current need and vulnerability; it does not estimate expected treatment benefit, preventable risk, or prognosis.

## 2. Materials and Methods

### 2.1. Study Design and Setting

This observational methodological study used anonymized routine-care data from 124 older adults living in 10 residential care facilities in Valencia, Spain. Data were collect-ed between June 2022 and April 2024. COVID-19 is treated only as a temporal context because outbreak status, vaccination, staffing disruption, menu modification, isolation, resident infection, and post-COVID symptoms were not systematically recorded. The study developed a preliminary priority score and did not evaluate longitudinal clinical outcomes.

### 2.2. Participants and Data Sources

The analytical cohort comprised 124 consenting residents from 10 pragmatically participating long-term care facilities. Inclusion required residence in a participating facility and sufficient routine-care information to calculate the nutritional-intake/anthropometric domain and at least one additional domain. The facilities were not randomly selected, and consecutive inclusion could not be verified. The available source records did not permit reconstruction of the total number screened, reasons for exclusion, palliative-care status, enteral feeding, recent hospitalization, acute infection or inflammatory status, or the precise consent pathway for residents with cognitive impairment. Consequently, a conventional participant-flow diagram could not be reconstructed; the analytical flow was 124 available eligible records and 124 records included. The dataset included demographic variables, centre of residence, sex, age, Barthel Index, Mini-Examination of Cognition (MEC), Tinetti score, Norton scale, observed weekly intake, MNA-SF, body mass index (BMI), previous and current weight, and biochemical variables including total proteins, albumin, transferrin, lipid profile, creatinine, uric acid, lymphocytes, vitamin D, iron, vitamin B12, and folate. Supplement recording and menu-choice information were also available. The Barthel Index, MEC, Tinetti score, and MNA-SF were interpreted according to established geriatric assessment literature [3,4,14,15,16]. Observed intake data were obtained from weekly menu intake records. In addition to the global weekly intake percentage, meal-component records were extracted for lunch, dinner, dessert, snack, bedtime snack, water and supplement-related items where available. These component-level data were used descriptively and to support interpretation of menu responsiveness. Component-level intake descriptors were meal component, valid *n* and mean ± SD.

### 2.3. Development of the VIVA Nutritional Intervention Priority Index

The VIVA Nutritional Intervention Priority Index (VIVA-NIPI) was constructed as a prespecified weighted multidomain score (Table 1). Development comprised variable selection, risk-oriented normalization, investigator-defined domain and subcriterion weights, proportional reweighting of available information, and resident-level calculation. VIVA (Vulnerability Index for Valencia Institutionalized Adults) was created for the present score and was not the name of a pre-existing study. No formal expert pairwise comparison or Delphi exercise was conducted. Each variable was transformed to a risk-oriented 0–100 score using pragmatic bounded mappings rather than diagnostic thresholds. Age was bounded between 65 and 95 years; albumin between 4.0 and 3.0 g/dL; transferrin between 250 and 150 mg/dL; vitamin B12 between 300 and 150 pg/mL; folate between 4.0 and 2.0 ng/mL; vitamin D between 30 and 10 ng/mL; and iron between 60 and 20 µg/dL. Values beyond each range were clipped to 0 or 100 to prevent extreme observations from dominating. BMI risk was defined as min [100, (22-BMI)/7 × 100] for BMI < 22 kg/m^2^, 0 for BMI 22–30 kg/m^2^, and min [50, (BMI-30)/10 × 50] for BMI > 30 kg/m^2^. These transformations require external calibration.

Worked example: for a hypothetical resident aged 85 years, Barthel 50, Tinetti 14, MEC 28, MNA-SF 9, intake 70%, BMI 21 kg/m^2^, albumin 3.2 g/dL, and total proteins 5.8 g/dL, the normalized component scores produce domain scores of 47.7, 29.1, and 64.8 and an overall score of 42.2/100.

The three domains were functional–cognitive vulnerability (0.30), nutritional-intake/anthropometric risk (0.45), and selected biochemical indicators relevant to nutritional assessment (0.25). The biochemical label was changed because albumin, total proteins, transferrin, iron, vitamin D, vitamin B12, and folate are affected by inflammation, hydration, renal and liver function, supplementation, acute illness, and comorbidity. Laboratory measurements originated from routine care rather than a standardized study protocol. Nutritional supplements were recorded for 8/124 residents (6.5%). Weights were prespecified clinical assumptions and not empirically optimized. The nutritional-intake/anthropometric domain received the largest nominal weight (0.45) because MNA-SF, current intake, and BMI represent the most proximal and potentially modifiable information for nutritional review. Functional–cognitive vulnerability received 0.30 because dependency, mobility, and cognition determine access to food, need for eating assistance, and feasibility of intervention. Selected biochemical indicators received 0.25 because they may add contextual information but are nonspecific and vulnerable to confounding by disease and laboratory availability. Within domains, Barthel and Tinetti were emphasized because dependency and mobility directly affect meal access; MNA-SF and observed intake were emphasized because they capture complementary screening and actual consumption information; and albumin and total proteins received the largest biochemical subweights because they were the most complete protein-related measures. These weights remain prespecified clinical assumptions rather than empirically optimized or consensus-derived values. The original reciprocal-matrix representation followed established multicriteria and AHP methodological literature [17,18], although its consistency is expected because the matrices were generated algebraically from prespecified weights.

Across residents, the median number of available score variables was 11 (range 7–14). Median availability was 4/4 functional–cognitive variables, 3/3 nutritional-intake/anthropometric variables, and 4/7 biochemical variables. Within the nominal 0.25 biochemical contribution, the effective overall albumin weight ranged from 0.075 to 0.250 (median 0.094), and the total-protein weight from 0.063 to 0.250 (median 0.076). Proportional reweighting therefore generates resident-specific score versions and limits strict comparability. A complete-case analysis across all score variables would have retained only a small, clinically selected subset because micronutrient testing was incomplete. Multiple imputation was not used because the dataset lacked sufficiently complete auxiliary clinical variables to support a defensible missing-at-random assumption. Accordingly, the primary proportional-reweighting analysis was complemented by high-completeness and no-biochemical-domain sensitivity analyses, and the resulting limitation is explicitly acknowledged.

### 2.4. Internal Construct Concordance Endpoints

Internal construct concordance was assessed using outcomes generated from, or directly overlapping with, score components. The exploratory k-means phenotype included age, Barthel Index, MEC, Tinetti score, MNA-SF, BMI risk, total proteins, and albumin and is therefore not an independent reference standard. Variables were median-imputed and z-standardized. K-means used k-means++ initialization, 50 random starts, and random_state = 42. Values of k = 2–5 were inspected; silhouette coefficients were 0.230, 0.168, 0.179, and 0.176. Three descriptive clusters contained 34, 53, and 37 residents. Because k = 2 had the highest silhouette value and bootstrap and centre-adjusted stability were not established, the three-cluster solution is exploratory only.

MNA-SF ≤ 11, weekly observed intake < 75%, albumin < 3.5 g/dL, and supplement re-cording were retained only as descriptive component-based coherence checks. No independent prospective outcome or blinded clinical adjudication was available. Accordingly, these analyses cannot establish clinical validity, diagnostic performance, prognosis, treatment response, or incremental clinical utility.

### 2.5. Statistical Analysis

Continuous variables were assessed graphically using histograms and quantile plots and summarized by mean ± standard deviation or median (interquartile range), as ap-propriate. The score was analyzed primarily as continuous. Lower, middle, and upper sample tertiles were used only for descriptive visualization and are not clinical priority categories. It is descriptive because its variables contribute directly to score construction; inferential *p* values were removed and epsilon-squared values are retained only as descriptive effect sizes. AUC confidence intervals used 2000 outcome-stratified percentile bootstrap resamples; records missing the relevant endpoint were excluded for that analysis. Confidence intervals were resident-level and did not account for centre clustering. Sparse outcomes were interpreted cautiously. Sensitivity analyses excluded age, excluded MNA-SF, used equal weights, retained only albumin and total proteins in the biochemical domain, and omitted that domain. Analyses were rerun using Python version 3.13.5 (Python Software Foundation, Wilmington, DE, USA), together with NumPy version 2.3.5, SciPy version 1.17.0, pandas version 2.2.3, scikit-learn version 1.8.0, and statsmodels version 0.14.6. A reproducible analytical code is provided as a Supplementary Material.

### 2.6. Ethics Approval

The study was approved by the Research Ethics Committee of the University of Valencia (reference: 1640722). Participation consent was obtained according to the approved procedures, and data were anonymized before analysis. The specific consent pathway for residents with cognitive impairment should be verified against the original ethics documentation. Reporting was checked against the complete STROBE checklist and relevant TRIPOD items for transparent reporting of model development, while recognizing that the present score is not a validated prediction model.

## 3. Results

### 3.1. Resident Characteristics and Data Availability

The analytical sample included 124 residents from 10 centres; 73 were women (58.9%). Mean age was 82.2 ± 10.1 years. Functional status was heterogeneous, with a mean Barthel Index of 68.8 ± 31.3 and a mean Tinetti score of 19.2 ± 9.0. Mean MEC was 29.4 ± 8.4, and mean MNA-SF was 11.2 ± 2.4. Observed weekly intake was available for 122 residents and averaged 88.2 ± 10.7% (Table 2).

Biochemical data showed a moderately preserved mean protein profile but substantial interindividual variability. Albumin was available for 118 residents and averaged 3.6 ± 0.4 g/dL, while total proteins were available for 115 residents and averaged 6.2 ± 0.6 g/dL. Micronutrient data were less complete, especially vitamin B12 and folate, which motivated proportional reweighting within the biochemical domain rather than complete-case exclusion.

### 3.2. Construction and Distribution of the VIVA-NIPI

**Figure 1:**
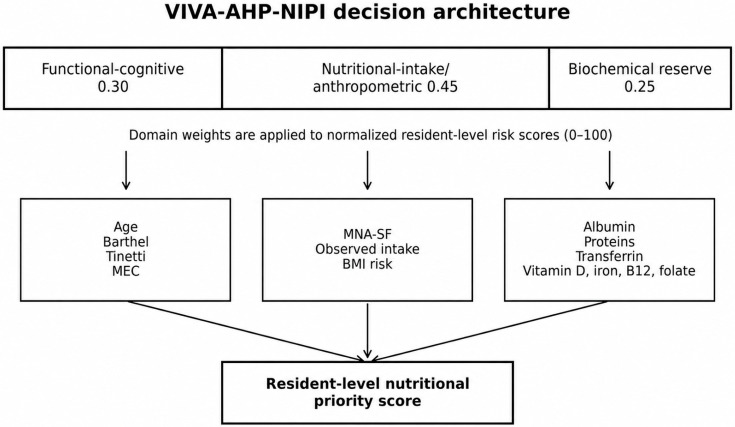
Weighted multidomain architecture of the VIVA-NIPI. The three domains and investigator-defined weights are shown explicitly. The structure is transparent but was not derived through a formal expert pairwise-comparison exercise.

The VIVA-NIPI was calculable for all 124 residents and had a mean of 24.4 ± 12.2 (range 2.3–55.7). The score is presented primarily as continuous. For descriptive visualization only, the lower, middle, and upper sample tertiles contained 42, 40, and 42 residents. These sample-dependent divisions necessarily classify approximately one-third of the cohort in the upper tertile and are not clinically validated thresholds. The weighting architecture is shown in Figure 1.

**Figure 2:**
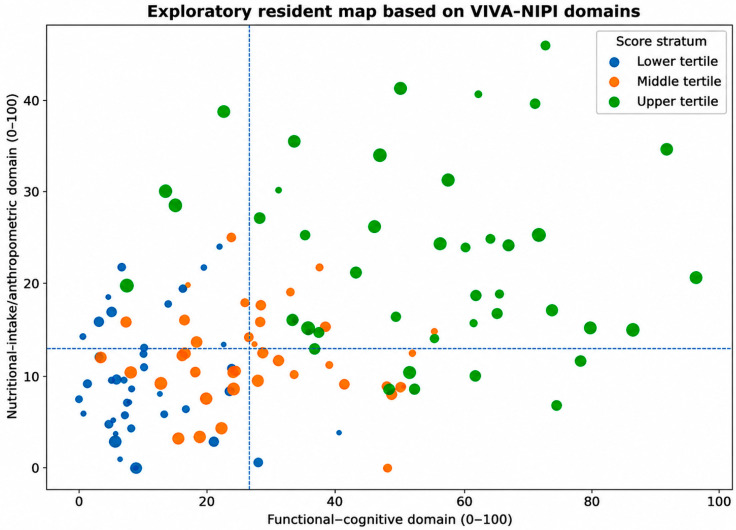
Exploratory resident map based on VIVA-NIPI domains. Each point represents one resident; dashed lines indicate sample medians, point area represents the selected biochemical-indicator score, and colours indicate sample tertiles only.

The exploratory resident map (Figure 2) displays the two main clinical domains, with point area proportional to the selected biochemical-indicator domain. Dashed lines indicate sample medians. Colours represent lower, middle, and upper sample score tertiles and do not represent clinical thresholds.

### 3.3. Profiles Across Priority Groups

Table 3 describes variables used in score construction across lower, middle, and upper score tertiles. The observed gradients are expected partly by construction and do not constitute independent validity evidence. Effect sizes are descriptive only.

**Figure 3:**
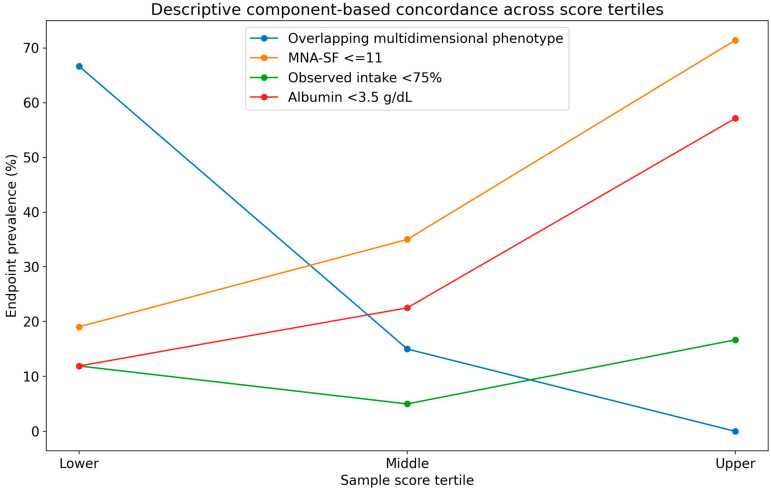
Descriptive component-based concordance across lower, middle, and upper score tertiles. The displayed endpoints overlap with score components and are not independent reference standards.

Component-based endpoint prevalence across score tertiles is shown in Figure 3. Be-cause the phenotype and secondary endpoints overlap with score components, these patterns demonstrate descriptive construct concordance rather than independent validation.

### 3.4. Internal Construct Concordance

The VIVA-NIPI showed an AUC of 0.918 (95% bootstrap CI 0.870–0.958) for the partly overlapping multidimensional phenotype. AUCs were 0.769 (0.680–0.853) for MNA-SF ≤ 11, 0.602 (0.450–0.750) for observed intake < 75%, 0.782 (0.688–0.867) for albumin < 3.5 g/dL, and 0.755 (0.575–0.909) for supplement recording. These values quantify internal concordance only. The low-intake and supplement outcomes were sparse (14 and eight cases), and their estimates are unstable. These results are shown in Table 4.

**Figure 4:**
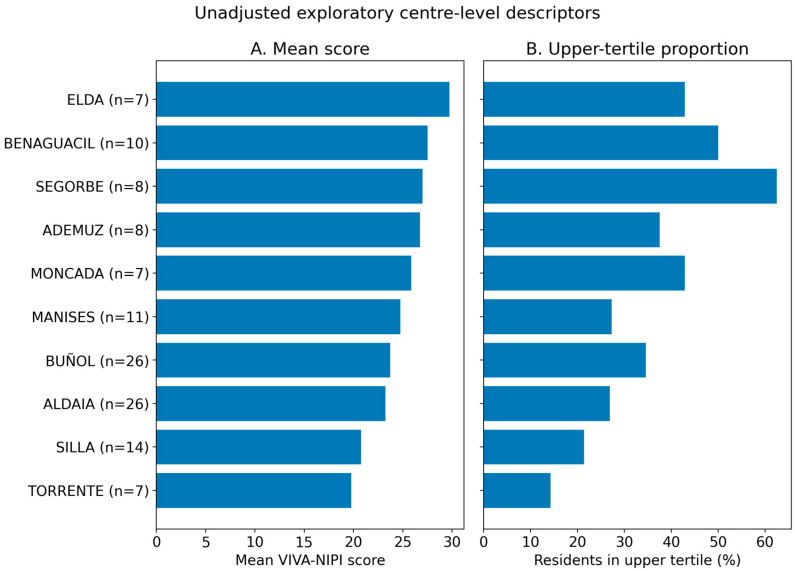
Unadjusted exploratory centre-level descriptors. Panel (**A**) shows the mean continuous VIVA-NIPI score and Panel (**B**) shows the percentage of residents in the upper sample tertile. Centre sample sizes are included in the labels. No case-mix adjustment, multilevel modelling, or cluster-robust inference was performed; the figure must not be interpreted as a quality comparison between centres.

Unadjusted centre-level variation in mean VIVA-NIPI scores and upper-tertile prevalence is shown descriptively in Figure 4. Centre sizes ranged from seven to 26 residents. The current sample was insufficient for reliable multilevel estimation or internal–external validation, and the bootstrap intervals were not cluster-robust. These results must not be interpreted as quality comparisons.

### 3.5. Sensitivity Analyses

Sensitivity analyses showed that removing age produced Spearman ρ = 0.991 and 40/42 (95.2%) upper-tertile overlap; removing MNA-SF produced ρ = 0.960 and 38/42 (90.5%) overlap; an equal-weight unweighted additive version produced ρ = 0.957 and 38/42 (90.5%) overlap; restricting biochemical information to albumin and total proteins produced ρ = 0.983 and 39/42 (92.9%) overlap; and omitting the biochemical domain produced ρ = 0.901 and 34/42 (81.0%) overlap. Component correlations indicated partial redundancy: MNA-SF correlated 0.384 with Barthel, 0.302 with Tinetti, 0.292 with BMI, 0.163 with MEC, and 0.034 with intake; Barthel and Tinetti correlated 0.762, and albumin and total proteins correlated 0.536. These analyses demonstrate rank stability under several assumptions but do not establish incremental clinical value over MNA-SF or simpler scores. The original alternative-weight scenarios were also retained for completeness: equal weights (rho = 0.991; 92.9% upper-tertile overlap), functional–cognitive-dominant weights (rho = 0.978; 88.1%), nutritional-intake-dominant weights (rho = 0.991; 95.2%), biochemical-indicator-dominant weights (rho = 0.972; 92.9%), and reduced biochemical influence (rho = 0.960; 90.5%). These values demonstrate numerical ranking stability only and do not establish clinical superiority of the selected weighting structure.

## 4. Discussion

This study developed a prespecified weighted multidomain nutritional priority score and assessed internal construct concordance and ranking stability. It is not a formal AHP model, a clinically validated triage tool, a diagnostic test, or a prognostic model. The overlapping AUC analyses primarily demonstrate mathematical concordance.

The observed cohort profile confirms substantial heterogeneity in functional performance, mobility, MNA-SF, intake, and selected biochemical indicators. This heterogeneity motivates research on transparent prioritization but does not validate the weighting structure or define intervention thresholds. This multidimensional heterogeneity is consistent with contemporary reviews of malnutrition in older adults [19,20]. The score is conceptually positioned after screening and assessment. MNA-SF identifies nutritional risk and GLIM supports diagnosis, whereas the VIVA-NIPI is intended only to organize the order of comprehensive review. It does not replace either instrument or clinical judgement. This positioning is consistent with current ESPEN guidance and the GLIM consensus framework [21,22]. Differences across score tertiles were expected because the compared variables contribute directly to score construction. They illustrate how the score aggregates information but must not be interpreted as independent clinical validation or as evidence that upper-tertile residents should automatically receive a particular intervention.

Observed intake contributes information that is not fully captured by MNA-SF in this dataset; their Spearman correlation was 0.034. Nevertheless, intake is only one component of the score, and the modest AUC for intake < 75% indicates that the score should not be interpreted as a direct detector of low intake. The importance of improving food and fluid intake through multiprofessional long-term-care strategies has also been emphasized previously [23,24].

Selected biochemical indicators contributed complementary information, but they do not constitute a direct measure of nutritional reserve. Albumin and related markers are influenced by inflammation, hydration, renal and liver function, disease burden, supplementation, and testing practices. Their availability may itself reflect clinical concern or centre practice, and proportional reweighting can increase the effective contribution of albumin and total proteins when other tests are absent. The explicit weighting architecture makes value judgements visible but does not establish benefit over simpler alternatives. The high rank correlation with the equal-weight score (ρ = 0.957) and the score excluding MNA-SF (ρ = 0.960) indicates that future work must demonstrate incremental value using independent clinical judgment, prospective outcomes, reclassification, net benefit, workload, and equity analyses. Because all available outcomes were overlapping constructs rather than an independent clinical endpoint, the study could not validly compare discrimination, reclassification, net benefit, or clinical utility of VIVA-NIPI versus MNA-SF alone, observed intake alone, or MNA-SF plus intake. The no-MNA-SF and equal-weight analyses therefore assess ranking dependence, not incremental clinical value. Multicriteria and AHP literature remains relevant as methodological background for transparent weighting [11,12,25,26], but the present score was not developed through a formal expert pairwise-comparison exercise. A future multidisciplinary elicitation study should report panel composition, pairwise judgments, aggregation, disagreement, consistency, and final consensus weights. Related applications in ageing, housing, therapeutic environments, and culturally tailored health interventions illustrate the broader use of structured multicriteria approaches [27,28,29]. The internal coherence results show that the score behaves in the expected direction relative to related constructs. They should not be interpreted as independent diagnostic accuracy because MNA-SF and albumin are index components and the vulnerability phenotype was generated from overlapping variables. The strongest AUC therefore represents construct coherence within this dataset, whereas the AUC of 0.602 for low intake demonstrates limited discrimination for a more specific intake endpoint.

Sensitivity analyses showed that ranking was reasonably stable after removing age or MNA-SF and under equal weighting, but omitting the biochemical domain changed eight of 42 upper-tertile classifications. These findings support transparency about model dependence rather than a claim of robustness sufficient for implementation.

Centre-level results are unadjusted exploratory descriptors, not quality comparisons. Facility selection, case mix, laboratory testing, documentation, staffing, food services, and missingness may differ between centres and preclude benchmarking.

### 4.1. Strengths and Limitations

Strengths include the use of real-world residential-care data, integration of observed intake with functional, cognitive, anthropometric, screening, and biochemical information, transparent weighting assumptions, continuous resident-level scoring, and multiple sensitivity analyses. The principal contribution is a reproducible research framework for studying nutritional-review prioritization, not a clinically validated decision rule. Several limitations materially restrict interpretation. The sample of 124 residents was a pragmatic regional cohort without an a priori sample-size calculation and was small for clustering, sparse-outcome regression, centre-level modelling, repeated sensitivity analyses, and threshold development. Recruitment flow, reasons for exclusion, acute clinical status, comorbidities, standardized laboratory conditions, and consent procedures for cognitive impairment could not be reconstructed fully. Missing biochemical data produced resident-specific effective weights and may reflect centre practice or clinical suspicion. Component redundancy, incorporation bias, lack of cluster-robust inference, and absence of an independent endpoint prevent claims of clinical validation. Complete-case analysis would be highly selective, while multiple imputation would depend on untestable missing-at-random assumptions with the available covariates.

Data collection occurred from June 2022 to April 2024. COVID-19 wording was removed from the title because pandemic-related exposures were not systematically measured and cannot be incorporated analytically.

### 4.2. Implications for Research and Practice

The next research step should use blinded multidisciplinary adjudication of resident profiles and/or prospective clinical outcomes. The panel should include relevant long-term care professionals, use prespecified urgency criteria, and report inter-rater reliability, consensus rankings, agreement with the VIVA-NIPI, and incremental value beyond MNA-SF and an unweighted score. Formal stakeholder weight elicitation, centre-aware validation, and equity analysis should follow. Prospective intervention research in nursing-home residents also supports evaluating whether structured nutritional action can preserve functional outcomes [30]. At this stage, the score should remain a research construct. Future studies must specify the user and review time horizon, actionable intervention, intended outcome, capacity constraints, and safeguards against age-related inequity. Clinically meaningful cutoffs should be derived against blinded expert judgement or prospective outcomes and externally calibrated rather than defined by sample tertiles. Future calibration should additionally account for setting-specific variation in MNA-defined nutritional status among older populations [31,32].

## 5. Conclusions

The VIVA-NIPI is a preliminary weighted multidomain score for organizing nutritional review in institutionalized older adults. The present study demonstrates internal construct concordance and descriptive rank stability only. It does not establish clinical validity, incremental value over MNA-SF or an unweighted score, prognostic performance, treatment benefit, or clinically actionable thresholds. Independent multidisciplinary adjudication or prospective outcomes, formal weight elicitation, centre-aware external validation, missing-data modelling, and equity assessment are required before implementation.

## Figures and Tables

**Table 1:** Domains, subcriteria, and prespecified weights used to calculate the VIVA-NIPI.

Domain	Domain Weight	Subcriterion	Subweight
Functional–cognitive vulnerability	0.30	Age	0.10
		Barthel Index	0.35
		Tinetti score	0.35
		MEC	0.20
Nutritional-intake/anthropometric risk	0.45	MNA-SF	0.40
		Weekly observed intake	0.40
		BMI risk	0.20
Selected biochemical indicators relevant to nutritional assessment	0.25	Albumin	0.30
		Total proteins	0.25
		Transferrin	0.15
		Vitamin D	0.10
		Iron	0.08
		Vitamin B12	0.07
		Folate	0.05

**Table 2:** Resident characteristics and variable availability.

Variable	Valid n	Mean ± SD	Median (IQR)
Age, years	116	82.2 ± 10.1	—
Barthel Index	118	68.8 ± 31.3	—
MEC cognitive score	114	29.4 ± 8.4	—
Tinetti score	117	19.2 ± 9.0	—
Norton scale	17	17.1 ± 3.4	—
Weekly observed intake, %	122	88.2 ± 10.7	—
MNA-SF	122	11.2 ± 2.4	—
BMI, kg/m^2^	124	28.0 ± 6.1	—
Total proteins, g/dL	115	6.2 ± 0.6	—
Albumin, g/dL	118	3.6 ± 0.4	—
Transferrin, mg/dL	72	207.6 ± 53.6	—
Creatinine, mg/dL	117	1.4 ± 3.7	0.90 (0.74–1.15)
Vitamin D, ng/mL	74	24.8 ± 15.8	—
Iron, μg/dL	83	65.1 ± 30.6	—
Vitamin B12, pg/mL	59	339.4 ± 158.1	—
Folate, ng/mL	55	7.5 ± 8.3	4.70 (3.50–8.85)

**Table 3:** Descriptive clinical and domain profiles across VIVA-NIPI score tertiles.

Variable	Lower Tertile	Middle Tertile	Upper Tertile	Valid *n* (L/M/U)	Epsilon^2^
VIVA-NIPI	12.2 ± 5.3	23.1 ± 2.6	38.7 ± 7.3	42/40/42	—
Functional–cognitive domain	11.8 ± 10.0	28.1 ± 13.4	53.8 ± 21.0	42/40/42	—
Nutritional-intake/anthropometric domain	9.9 ± 6.6	13.1 ± 6.0	23.8 ± 10.2	42/40/42	—
Selected biochemical-indicator domain	16.7 ± 14.8	35.7 ± 16.3	48.0 ± 15.8	42/40/42	—
Age, years	76.8 ± 9.7	83.7 ± 9.8	85.8 ± 8.6	39/37/40	0.111
Barthel Index	94.2 ± 9.2	72.0 ± 22.0	40.5 ± 29.9	41/37/40	0.520
Tinetti score	25.3 ± 3.9	20.8 ± 6.7	11.2 ± 8.9	41/38/38	0.418
MEC	30.6 ± 4.0	31.2 ± 10.4	26.4 ± 9.3	42/35/37	0.023
Weekly intake, %	89.6 ± 10.2	90.4 ± 8.6	84.8 ± 12.3	40/40/42	0.038
MNA-SF	12.5 ± 1.4	11.7 ± 1.4	9.5 ± 2.8	41/39/42	0.260
BMI, kg/m^2^	27.6 ± 4.5	29.6 ± 5.2	26.9 ± 7.9	42/40/42	0.011
Total proteins, g/dL	6.6 ± 0.5	6.1 ± 0.5	5.9 ± 0.5	38/38/39	0.307
Albumin, g/dL	3.9 ± 0.4	3.6 ± 0.3	3.4 ± 0.3	39/38/41	0.332
Vitamin D, ng/mL	23.6 ± 12.8	25.2 ± 21.0	25.4 ± 12.5	24/25/25	0.000
Vitamin B12, pg/mL	293.7 ± 103.5	399.0 ± 163.3	323.1 ± 182.6	17/22/20	0.028

**Table 4:** Internal construct concordance analyses for the VIVA-NIPI.

Endpoint	Cases/Total	AUC (95% CI)	OR per 10-Point Increase (95% CI)
High multidimensional vulnerability phenotype	34/124	0.918 (0.870–0.958)	8.34 (3.88–17.94)
MNA-SF ≤ 11	52/124	0.769 (0.680–0.853)	2.50 (1.69–3.70)
Weekly observed intake < 75%	14/124	0.602 (0.450–0.750)	1.34 (0.86–2.08)
Albumin < 3.5 g/dL	38/124	0.782 (0.688–0.867)	2.68 (1.78–4.06)
Supplement recorded in routine care	8/124	0.755 (0.575–0.909)	2.21 (1.20–4.07)

## Data Availability

The original contributions presented in this study are included in the article/Supplementary Materials. Further inquiries can be directed to the corresponding author.

## Supplementary Materials

The following supporting information can be downloaded at https://www.mdpi.com/article/10.3390/nu18152533/s1, Table S1: Variable coding and normalization rules; Table S2: Original reciprocal matrices and prespecified weights; Table S3: Extended data availability; Table S4: Internal construct concordance endpoint rationale; Table S5: Complete STROBE checklist with manuscript page and line references; Table S6: Applicable TRIPOD reporting items; Table S7: Component-based endpoint prevalence across score tertiles; Table S8: Centre-level descriptive results; Table S9: Sensitivity analyses, including a base-scenario overlap of 42/42 (100.0%); Table S10: Spearman component correlation matrix.

## Abbreviations

The following abbreviations are used in this manuscript:

AHP	Analytic Hierarchy Process
AUC	Area Under the receiver operating characteristic Curve
BMI	Body Mass Index
CI	Confidence Interval
GLIM	Global Leadership Initiative on Malnutrition
MEC	Mini-Examination of Cognition
MNA-SF	Mini Nutritional Assessment-Short Form
NIPI	Nutritional Intervention Priority Index
OR	Odds ratio
VIVA	Vulnerability Index: Valencia institutionalized Adults
