# Supplementary material for: A Weighted Multidomain Score for Prioritizing Nutritional Review in Institutionalized Older Adults: Development and Internal Assessment
Source: Nutrients. 2026 Aug 4;18(15):2533. doi: 10.3390/nu18152533 (PMC13468573; doi:10.3390/nu18152533)
Supplement: Supplementary file 1 [file nutrients-18-02533-s001.zip › nutrients-4439104-supplementary.pdf]

Supplementary Materials

A Weighted Multidomain Score for Prioritizing Nutritional Review in Institutionalized Older Adults: Development and Internal Assessment

Elena Moreno-Guillamont, Carmen I. Sáez-Lleó, Maria Auxiliadora Dea-Ayuela, and Jose M. Soriano

Supplementary Table S1. Variable coding and normalization rules

Variable	Domain	Normalization rule	Interpretation and limitation
Age	Functional-cognitive	100 × (age − 65)/30, clipped to 0–100	Pragmatic 65–95-year anchor; higher age increases review priority; equity sensitivity analysis excludes age.
Barthel Index	Functional-cognitive	100 − Barthel	Lower functional independence increases priority.
Tinetti score	Functional-cognitive	100 × (28 − Tinetti)/28	Lower mobility and balance increase priority.
MEC	Functional-cognitive	100 × (35 − MEC)/35	Lower cognitive score increases priority.
MNA-SF	Nutritional-intake/anthropometric	100 × (14 − MNA-SF)/14	Lower nutritional screening score increases priority.
Observed weekly intake	Nutritional-intake/anthropometric	100 − observed intake (%)	Lower actual intake increases priority.
BMI	Nutritional-intake/anthropometric	BMI <22: min[100, 100×(22−BMI)/7]; BMI 22–30: 0; BMI >30: min[50, 50×(BMI−30)/10]	Bidirectional mapping; low BMI receives greater maximum contribution than high BMI.
Albumin	Selected biochemical indicators	100 × (4.0 − albumin)/1.0, clipped to 0–100	Pragmatic 4.0–3.0 g/dL anchor; nonspecific and affected by inflammation/hydration.
Total proteins	Selected biochemical indicators	100 × (6.5 − proteins)/1.5, clipped to 0–100	Pragmatic 6.5–5.0 g/dL anchor.
Transferrin	Selected biochemical indicators	100 × (250 − transferrin)/100, clipped to 0–100	Pragmatic 250–150 mg/dL anchor.
Vitamin D	Selected biochemical indicators	100 × (30 − vitamin D)/20, clipped to 0–100	Pragmatic 30–10 ng/mL anchor.
Iron	Selected biochemical indicators	100 × (60 − iron)/40, clipped to 0–100	Pragmatic 60–20 µg/dL anchor.
Vitamin B12	Selected biochemical indicators	100 × (300 − B12)/150, clipped to 0–100	Pragmatic 300–150 pg/mL anchor.
Folate	Selected biochemical indicators	100 × (4.0 − folate)/2.0, clipped to 0–100	Pragmatic 4.0–2.0 ng/mL anchor.

Worked example. For a hypothetical resident aged 85 years, Barthel 50, Tinetti 14, MEC 28, MNA-SF 9, intake 70%, BMI 21 kg/m², albumin 3.2 g/dL, and total proteins 5.8 g/dL, the component risk scores are 66.7, 50.0, 50.0, 20.0, 35.7, 30.0, 14.3, 80.0, and 46.7. After applying the prespecified subweights and proportional reweighting of the two available biochemical indicators, the domain scores are 47.7, 29.1, and 64.8, yielding an overall score of 42.2/100.

Supplementary Table S2. Original reciprocal matrices and prespecified weights

These matrices were generated algebraically from investigator-prespecified weights and are reported for transparency only. Perfect consistency is expected by construction and is not evidence of methodological validity.

S2A. Main domains

Criterion	Functional-cognitive	Nutritional-intake/anthropometric	Selected biochemical indicators
Functional-cognitive	1.000	0.667	1.200
Nutritional-intake/anthropometric	1.500	1.000	1.800
Selected biochemical indicators	0.833	0.556	1.000

S2B. Functional-cognitive subcriteria

Criterion	Age	Barthel	Tinetti	MEC
Age	1.000	0.286	0.286	0.500
Barthel	3.500	1.000	1.000	1.750
Tinetti	3.500	1.000	1.000	1.750
MEC	2.000	0.571	0.571	1.000

S2C. Nutritional-intake/anthropometric subcriteria

Criterion	MNA-SF	Observed intake	BMI
MNA-SF	1.000	1.000	2.000
Observed intake	1.000	1.000	2.000
BMI	0.500	0.500	1.000

S2D. Selected biochemical indicators

Criterion	Albumin	Total proteins	Transferrin	Vitamin D	Iron	Vitamin B12	Folate
Albumin	1.000	1.200	2.000	3.000	3.750	4.286	6.000
Total proteins	0.833	1.000	1.667	2.500	3.125	3.571	5.000
Transferrin	0.500	0.600	1.000	1.500	1.875	2.143	3.000
Vitamin D	0.333	0.400	0.667	1.000	1.250	1.429	2.000
Iron	0.267	0.320	0.533	0.800	1.000	1.143	1.600
Vitamin B12	0.233	0.280	0.467	0.700	0.875	1.000	1.400
Folate	0.167	0.200	0.333	0.500	0.625	0.714	1.000

Matrix	n	λmax	Consistency index	Consistency ratio
Main domains	3	3.000	0.000	0.000
Functional-cognitive subcriteria	4	4.000	0.000	0.000
Nutritional-intake/anthropometric subcriteria	3	3.000	0.000	0.000
Selected biochemical indicators	7	7.000	0.000	0.000

Supplementary Table S3. Extended data availability

Variable	Valid n	Missing n	Completeness
Age, years	116	8	93.5%
Barthel Index	118	6	95.2%
MEC cognitive score	114	10	91.9%
Tinetti score	117	7	94.4%
Norton scale	17	107	13.7%
Weekly observed intake, %	122	2	98.4%
MNA-SF	122	2	98.4%
BMI, kg/m²	124	0	100.0%
Total proteins, g/dL	115	9	92.7%
Albumin, g/dL	118	6	95.2%
Transferrin, mg/dL	72	52	58.1%
Total cholesterol, mg/dL	120	4	96.8%
HDL cholesterol, mg/dL	108	16	87.1%
LDL cholesterol, mg/dL	91	33	73.4%
Triglycerides, mg/dL	108	16	87.1%
Creatinine, mg/dL	117	7	94.4%
Uric acid, mg/dL	97	27	78.2%
Lymphocytes, $\times 10^9$/L	115	9	92.7%
Vitamin D, ng/mL	74	50	59.7%
Iron, µg/dL	83	41	66.9%
Vitamin B12, pg/mL	59	65	47.6%
Folate, ng/mL	55	69	44.4%

Resident-level score availability: median 11 variables (range 7–14); functional-cognitive domain median 4/4, nutritional-intake/anthropometric domain median 3/3, and biochemical domain median 4/7. Effective overall albumin weight ranged from 0.075 to 0.250 (median 0.094), and effective total-protein weight ranged from 0.063 to 0.250 (median 0.076).

Supplementary Table S4. Internal construct-concordance endpoint rationale

Endpoint	Cases/total	Rationale	Interpretation limit	AUC (95% bootstrap CI)
High multidimensional vulnerability phenotype	34/124	Exploratory k-means construct combining age, Barthel, MEC, Tinetti, MNA-SF, BMI risk, total proteins, and albumin.	Substantial incorporation bias; not an independent reference standard.	0.918 (0.870–0.958)
MNA-SF ≤11	52/124	Established screening-level nutritional risk.	MNA-SF is a direct score component; descriptive coherence check only.	0.769 (0.680–0.853)

Endpoint	Cases/total	Rationale	Interpretation limit	AUC (95% bootstrap CI)
Observed weekly intake <75%	14/124	Immediate low-intake marker and potentially modifiable care target.	Sparse endpoint and intake-recording variability; modest discrimination.	0.602 (0.450–0.750)
Albumin <3.5 g/dL	38/124	Pragmatic biochemical indicator relevant to nutritional assessment.	Albumin is a direct component and is affected by inflammation, hydration, and disease.	0.782 (0.688–0.867)
Supplement recorded in routine care	8/124	Pragmatic indicator of prior nutritional concern or intervention.	Sparse and dependent on documentation and centre practice.	0.755 (0.575–0.909)

Supplementary Table S5. Complete STROBE checklist

Item	Checklist topic	Recommendation	Manuscript location
1	Title and abstract	Indicate the study design and provide an informative, balanced summary.	Title and Abstract, p. 1
2	Background/rationale	Explain the scientific background and rationale.	Introduction, pp. 2–3
3	Objectives	State specific objectives and prespecified hypotheses.	Introduction, final paragraph, p. 3
4	Study design	Present key elements of study design early.	Methods 2.1, p. 3
5	Setting	Describe setting, locations, and relevant dates.	Methods 2.1, p. 3
6	Participants	Give eligibility criteria and participant selection.	Methods 2.2, p. 4; Discussion 4.1, p. 16
7	Variables	Define outcomes, exposures, predictors, confounders, and diagnostic criteria.	Methods 2.2–2.4, pp. 4–7; Table 1; Supplementary Table S1
8	Data sources/measurement	Describe data sources and assessment methods.	Methods 2.2–2.3, pp. 4–6; Supplementary Tables S1–S3
9	Bias	Describe efforts to address potential bias.	Methods 2.2–2.5, pp. 4–7; Discussion 4.1, p. 16
10	Study size	Explain how study size was arrived at.	Methods 2.2, p. 4; Discussion 4.1, p. 16; pragmatic sample of 124 residents
11	Quantitative variables	Explain handling of quantitative variables and groupings.	Methods 2.3 and 2.5, pp. 4–7; Supplementary Table S1
12a	Statistical methods	Describe all statistical methods.	Methods 2.5, p. 7
12b	Subgroups/interactions	Describe subgroup and interaction methods.	Centre-level exploratory analysis in Results 3.4, pp. 12–14; Supplementary Table S8
12c	Missing data	Explain how missing data were addressed.	Methods 2.3 and 2.5, pp. 4–7; Supplementary Tables S3 and S9
12d	Sampling strategy	Describe analytical methods accounting for sampling strategy.	Not applicable: pragmatic facility sample; absence of centre-cluster adjustment stated in Methods 2.5 and Discussion 4.1
12e	Sensitivity analyses	Describe sensitivity analyses.	Methods 2.5, p. 7; Results 3.5, p. 14; Supplementary Table S9
13	Participants	Report numbers at each stage and reasons for non-participation.	Methods 2.2, p. 4: 124 available eligible records and 124 analysed; unavailable screening denominator stated
14	Descriptive data	Give participant characteristics and missingness.	Results 3.1, pp. 8–9; Table 2; Supplementary Table S3
15	Outcome data	Report numbers of outcome events or summary measures.	Results 3.4, pp. 12–14; Table 4; Supplementary Tables S4 and S7
16	Main results	Give unadjusted/adjusted estimates and precision.	Results 3.3–3.5, pp. 10–14; Tables 3–4; no adjusted causal estimates claimed
17	Other analyses	Report subgroup, interaction, and sensitivity analyses.	Results 3.4–3.5, pp. 12–14; Supplementary Tables S8–S10
18	Key results	Summarize key results with reference to objectives.	Discussion opening, pp. 14–15
19	Limitations	Discuss limitations, direction, and magnitude of bias.	Discussion 4.1, p. 16
20	Interpretation	Provide cautious interpretation considering multiplicity and external evidence.	Discussion, pp. 14–17; Conclusions, p. 17
21	Generalisability	Discuss external validity.	Discussion 4.1–4.2, pp. 16–17
22	Funding	State funding and role of funders.	Funding statement, p. 17: no external funding

Supplementary Table S6. Applicable TRIPOD reporting items

TRIPOD items are used here as a transparency aid for a decision-support score. VIVA-NIPI is not presented as a validated diagnostic or prognostic prediction model, and non-applicable items are identified explicitly.

Reporting item	Expected information	Location	Status/qualification
Title	Identify development/internal assessment and target population.	Title	Addressed
Abstract	Structured summary of objectives, data, methods, results, and limitations.	Abstract	Addressed
Background and objectives	Explain intended use, existing tools, and development objective.	Introduction	Addressed
Source of data	Describe routine-care source, dates, facilities, and design.	Methods 2.1–2.2	Addressed
Participants	Eligibility and selection process.	Methods 2.2	Partly addressed; screening denominator unavailable
Outcome/decision target	Define what the score is intended to prioritize.	Introduction and Methods	Addressed: near-term nutritional review urgency

Reporting item	Expected information	Location	Status/qualification
Predictors/components	Define all score components and timing.	Methods 2.2–2.3; Tables 1 and S1	Addressed
Sample size	Explain study-size basis.	Methods/Limitations	Pragmatic; no a priori calculation
Missing data	Describe missingness and handling.	Methods 2.3–2.5; Tables S3 and S9	Addressed with limitations
Statistical analysis	Describe score construction, normalization, weighting, and intervals.	Methods 2.3–2.5	Addressed
Model specification	Provide complete scoring procedure.	Table 1 and Supplementary Tables S1–S2	Addressed
Internal validation	Describe resampling and internal assessment.	Methods 2.5	Bootstrap CIs; not optimism-corrected clinical validation
Performance measures	Report discrimination/concordance measures and precision.	Results 3.4; Table 4	Addressed with incorporation-bias caveat
Risk groups/cutoffs	Explain creation and interpretation of groups.	Methods 2.5; Results 3.2	Tertiles descriptive only
Participant flow	Report numbers through analysis.	Methods 2.2	124 available and 124 analyzed; full flow unavailable
Participant characteristics	Describe cohort and missingness.	Results 3.1; Tables 2 and S3	Addressed
Model development results	Report component weights and score distribution.	Results 3.2; Table 1	Addressed
Model performance	Report internal construct concordance.	Results 3.4; Table 4	Addressed, not clinical validation
Model use	Explain how to calculate and intended use.	Methods 2.3; Table S1; worked example	Addressed
Limitations	Discuss overfitting, incorporation bias, missingness, clustering, and generalizability.	Discussion 4.1	Addressed
Interpretation	Interpret in relation to intended use and existing tools.	Discussion 4	Addressed conservatively
Implications	State need for external validation and clinical adjudication.	Discussion 4.2; Conclusions	Addressed

Supplementary Table S7. Component-based endpoint prevalence across score tertiles

Endpoint	Lower tertile	Middle tertile	Upper tertile	P value
High multidimensional vulnerability phenotype	0.0%	15.0%	66.7%	<0.001
MNA-SF ≤11	16.7%	37.5%	71.4%	<0.001
Observed weekly intake <75%	11.9%	5.0%	16.7%	0.246
Albumin <3.5 g/dL	9.5%	25.0%	57.1%	<0.001
Supplement recorded in routine care	2.4%	5.0%	11.9%	0.186

These gradients are descriptive and partly expected by construction because several endpoints are direct components of, or overlap with, the score. P values are not evidence of independent validation.

Supplementary Table S8. Centre-level descriptive results

Centre	n	Mean VIVA-NIPI	Upper tertile (%)	Mean intake (%)	Mean MNA-SF
ELDA	7	30.0	42.9	84.1	9.6
BENAGUACIL	10	28.2	50.0	90.5	11.9
SEGORBE	8	27.6	62.5	88.7	11.5
ADEMUZ	8	27.0	37.5	82.3	11.2
MONCADA	7	26.2	42.9	86.3	11.0
MANISES	11	25.3	27.3	88.4	12.0
BUÑOL	26	24.1	34.6	91.6	9.4
ALDAIA	26	23.4	26.9	89.6	11.5
SILLA	14	21.0	21.4	82.2	12.5
TORRENTE	7	20.0	14.3	91.4	13.1

Unadjusted exploratory descriptors only. Differences may reflect case mix, documentation, testing practice, missingness, staffing, and food-service organization and must not be interpreted as quality comparisons.

Supplementary Table S9. Sensitivity analyses

Scenario	Weights/modification	Spearman ρ vs base	Upper-tertile overlap	Purpose
Base weighting scenario	0.30, 0.45, 0.25	1.000	42/42 (100.0%)	Reference
Equal domain weights	0.33, 0.33, 0.33	0.991	39/42 (92.9%)	Domain-weight scenario
Functional-cognitive dominant	0.45, 0.35, 0.20	0.978	37/42 (88.1%)	Domain-weight scenario
Nutritional-intake dominant	0.25, 0.55, 0.20	0.991	40/42 (95.2%)	Domain-weight scenario

Scenario	Weights/modification	Spearman ρ vs base	Upper-tertile overlap	Purpose
Biochemical-indicator dominant	0.25, 0.35, 0.40	0.972	39/42 (92.9%)	Domain-weight scenario
Reduced biochemical influence	0.35, 0.55, 0.10	0.960	38/42 (90.5%)	Domain-weight scenario
Age excluded	Reweighted functional-cognitive domain	0.991	40/42 (95.2%)	Equity sensitivity
MNA-SF excluded	Reweighted nutritional domain	0.960	38/42 (90.5%)	Redundancy sensitivity
Simple unweighted additive score	Equal domains and equal subcriteria	0.957	38/42 (90.5%)	Complexity comparison
Albumin and total proteins only	Biochemical domain restricted	0.983	39/42 (92.9%)	High-completeness sensitivity
Biochemical domain omitted	0.40, 0.60, 0.00	0.901	34/42 (81.0%)	Missingness/domain sensitivity

Overlap was defined as the number of residents shared with the 42-resident base upper-tertile group divided by 42. These analyses assess rank stability and do not establish clinical utility or superiority over simpler tools.

Supplementary Table S10. Spearman component correlation matrix

Pairwise complete observations were used, with a minimum of 10 valid pairs. Positive coefficients indicate that the raw variables increase together; because some components are reverse-coded in the score, these correlations should be interpreted as construct overlap rather than score-direction correlations.

Variable	Age	Barthel	Tinetti	MEC	MNA-SF	Observed intake	BMI	Albumin	Total proteins	Transferrin	Vitamin D	Iron	Vitamin B12	Folate
Age	1.000	-0.294	-0.351	-0.137	-0.025	-0.181	-0.161	-0.224	-0.207	-0.187	-0.034	-0.197	0.231	0.038
Barthel	-0.294	1.000	0.762	0.152	0.384	0.156	0.051	0.281	0.247	0.169	-0.157	0.095	-0.080	0.102
Tinetti	-0.351	0.762	1.000	0.058	0.302	0.228	0.168	0.225	0.161	0.119	-0.125	0.109	0.021	0.160
MEC	-0.137	0.152	0.058	1.000	0.163	0.015	-0.138	0.044	0.004	0.218	-0.040	0.026	0.015	-0.102
MNA-SF	-0.025	0.384	0.302	0.163	1.000	0.034	0.292	0.291	0.314	0.233	-0.177	-0.016	0.068	0.090
Observed intake	-0.181	0.156	0.228	0.015	0.034	1.000	0.113	-0.019	0.003	-0.254	0.016	0.180	0.309	0.390
BMI	-0.161	0.051	0.168	-0.138	0.292	0.113	1.000	0.002	0.062	-0.004	0.030	-0.077	0.037	0.050
Albumin	-0.224	0.281	0.225	0.044	0.291	-0.019	0.002	1.000	0.536	0.433	-0.053	0.283	-0.285	-0.073
Total proteins	-0.207	0.247	0.161	0.004	0.314	0.003	0.062	0.536	1.000	0.274	-0.013	0.180	-0.143	0.127
Transferrin	-0.187	0.169	0.119	0.218	0.233	-0.254	-0.004	0.433	0.274	1.000	0.104	0.261	-0.116	-0.032
Vitamin D	-0.034	-0.157	-0.125	-0.040	-0.177	0.016	0.030	-0.053	-0.013	0.104	1.000	0.155	0.196	-0.252
Iron	-0.197	0.095	0.109	0.026	-0.016	0.180	-0.077	0.283	0.180	0.261	0.155	1.000	0.142	0.255
Vitamin B12	0.231	-0.080	0.021	0.015	0.068	0.309	0.037	-0.285	-0.143	-0.116	0.196	0.142	1.000	0.048
Folate	0.038	0.102	0.160	-0.102	0.090	0.390	0.050	-0.073	0.127	-0.032	-0.252	0.255	0.048	1.000
